# Antioxidant defence system as a rational target for Chagas disease and Leishmaniasis chemotherapy

**DOI:** 10.1590/0074-02760210401

**Published:** 2022-02-28

**Authors:** Ana Maria Murta Santi, Silvane Maria Fonseca Murta

**Affiliations:** 1 Fundação Oswaldo Cruz-Fiocruz, Instituto René Rachou, Grupo de Genômica Funcional de Parasitos, Belo Horizonte, MG, Brasil

**Keywords:** Trypanosoma cruzi, *Leishmania* spp, chemotherapy, antioxidant defence, drug resistance

## Abstract

Chagas disease and leishmaniasis are neglected tropical diseases caused by the protozoan parasites *Trypanosoma cruzi* and *Leishmania* spp., respectively. They are among the most important parasitic diseases, affecting millions of people worldwide, being a considerable global challenge. However, there is no human vaccine available against *T. cruzi* and *Leishmania* infections, and their control is based mainly on chemotherapy. Treatments for Chagas disease and leishmaniasis have multiple limitations, mainly due to the high toxicity of the available drugs, long-term treatment protocols, and the occurrence of drug-resistant parasite strains. In the case of Chagas disease, there is still the problem of low cure rates in the chronic stage of the disease. Therefore, new therapeutic agents and novel targets for drug development are urgently needed. Antioxidant defence in Trypanosomatidae is a potential target for chemotherapy because the organisms present a unique mechanism for trypanothione-dependent detoxification of peroxides, which differs from that found in vertebrates. Cellular thiol redox homeostasis is maintained by the biosynthesis and reduction of trypanothione, involving different enzymes that act in concert. This study provides an overview of the antioxidant defence focusing on iron superoxide dismutase A, tryparedoxin peroxidase, and ascorbate peroxidase and how the enzymes play an important role in the defence against oxidative stress and their involvement in drug resistance mechanisms in *T. cruzi* and *Leishmania* spp.

Chagas disease and leishmaniasis are infectious, parasitic diseases caused by protozoan parasites of the Trypanosomatidae family. *Trypanosoma cruzi* is the etiological agent of Chagas disease (American trypanosomiasis), affecting 6-7 million people globally.^1^ It is endemic to 21 continental Latin American countries, and due to increased migration, the disease has spread across Europe, the United States, Canada, and Japan.^2^ Leishmaniasis are a complex of diseases caused by different species of parasites of the genus *Leishmania*, currently affecting 12 million people globally and presenting an incidence of 0.7-1.0 million new cases annually from nearly 100 endemic countries.^3^ The disease can comprise the following main clinical forms: cutaneous leishmaniasis, characterised by cutaneous and mucosal lesions, or visceral leishmaniasis (VL), in which the parasites have tropism for internal organs such as the liver and spleen.^4^ VL is the most severe form of the disease and can be lethal if left untreated.

No human vaccine is available for Chagas disease or leishmaniasis, and currently, few drugs are available to treat the diseases. Nifurtimox (5-nitrofuran; NFX) and benznidazole (2-nitroimidazole; BZ) have been used for Chagas disease chemotherapy. Although these drugs have been in use for more than 50 years, they have several drawbacks, including low cure rates in the chronic stage of the disease, significant toxic side effects, and the existence of naturally resistant strains of *T. cruzi*.^5^ Few drugs, including pentavalent antimonials (e.g., sodium stibogluconate and meglumine antimoniate), amphotericin B and formulations, miltefosine, paromomycin sulphate, and pentamidine isethionate^6^ are currently available for leishmaniasis treatment. Chemotherapy for leishmaniasis presents several problems, such as high drug toxicity, long treatment protocols, and the occurrence of drug-resistant parasite strains. Therefore, there is a need to understand drug resistance mechanisms and identify new molecular targets for drug development against Chagas disease and leishmaniasis. This article focuses on studies elucidating the importance of antioxidant defence against oxidative stress and its association with drug resistance mechanisms in *T. cruzi* and *Leishmania* spp. being considered as a rational target for chemotherapy against the important neglected tropical diseases.

*Antioxidant defence* - Trypanosomatids are frequently exposed to different reactive oxygen species (ROS), such as superoxide anions, hydrogen peroxide (H_2_O_2_), and hydroxyl radicals, produced by cellular metabolism and external agents, including products of the immune response of the host and drug metabolism.^7^ Since ROS can damage various cellular components, including membrane lipids, nucleic acids, and proteins, all organisms possess defence mechanisms based on antioxidant enzymes.^8^ However, trypanosomatid cells lack catalase, selenium-dependent glutathione peroxidase (GPX), glutathione reductase, and thioredoxin reductase.^7^ ^,^ ^9^ Instead, Trypanosomatids possess a peculiar antioxidant defence mechanism based on the low molecular mass dithiol trypanothione [bis(glutathionyl)spermidine; T(SH)_2_].^7^ ^,^ ^10^ ^,^ ^11^ ^,^ ^12^ The trypanothione is a central thiol that delivers electrons for the synthesis of DNA precursors, the detoxification of hydroperoxides, and other trypanothione-dependent pathways.^13^ Trypanothione directly reduces tryparedoxin, dehydroascorbate, and glutathione disulphide by sequential reactions coupled with the reductive detoxification of peroxides and the formation of deoxyribonucleotides (Figure). Trypanothione disulphide (TS_2_) is reduced by nicotinamide adenine dinucleotide phosphate to trypanothione T(SH)_2_ in a reaction catalysed by trypanothione reductase. Thus, cellular thiol redox homeostasis is maintained by biosynthesis and reduction of trypanothione.^13^

**Antioxidant defense:**
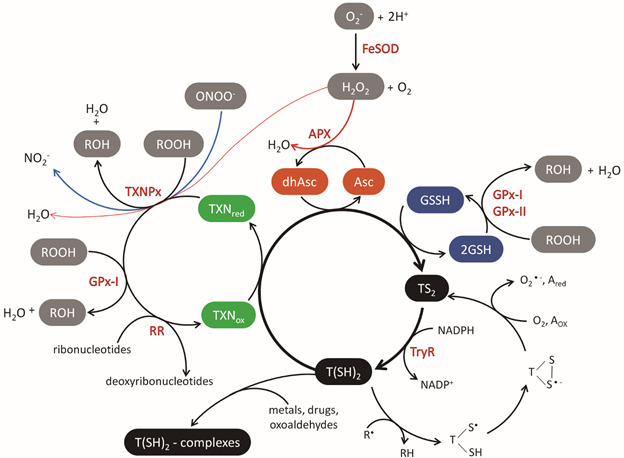
system in *Trypanosoma cruzi* and *Leishmania*. Iron-superoxide dismutases (FeSODs) protect parasites against superoxide radicals (O_2_^-^), which are converted to oxygen (O_2_) and hydrogen peroxide (H_2_O_2_). Tryparedoxin peroxidases (TXNPx) use tryparedoxin to detoxify H_2_O_2_, hydroperoxides (ROOH), and peroxynitrites (NOOH^-^). Ascorbate peroxidase (APX) converts H_2_O_2_ to water (H_2_O). Trypanothione reductase (TryR) is an enzyme that utilises NADPH to keep trypanothione in its reduced form T(SH)_2_. T(SH)_2_ converts tryparedoxin (TXN) to its reduced form, dehydroascorbate (dhAsc) to ascorbate (Asc), and glutathione disulphide (GSSG) to glutathione (GSH). The sequential reactions are coupled to the reductive detoxification of peroxides H_2_O_2_, ROOH, and NOOH^-^ and the synthesis of deoxyribonucleotides by ribonucleotide reductase (RR). Resistance to hydro- and lipid-hydroperoxides is conferred by non-selenium glutathione peroxidases-like (GPX-I and GPX-II), which utilise glutathione and/or tryparedoxin as reducing substrates. T(SH)_2_ can interact directly with different electrophiles in the detoxification of oxoaldehydes, metals, and drugs. T(SH)_2_ can react with radical species (R•) in scavenging and/or repair reactions, resulting in the production of trypanothione thiol radicals. The sulphur-centred radical is expected to combine with the vicinal thiol to produce a trypanothione disulphide anion radical, which changes to the stable trypanothione disulphide (TS_2_) with the formation of secondary radicals, including superoxide (O_2_^-^), which will be detoxified later. NO_2_, nitrite; A, one-electron oxidant. Figure adapted from Irigoin et al*.* ^7^

The defence machinery in trypanosomatids is composed of many enzymes distributed in diverse cellular compartments that are activated by various oxidants.^14^ Iron-superoxide dismutases (FeSODs) detoxify superoxide radicals (O_2_^−^) generated in the cytosol (FeSOD-B1), glycosomes (FeSOD-B1-2), and mitochondria (FeSOD-A and C), which convert to oxygen (O_2_) and hydrogen peroxide (H_2_O_2_) (Figure). H_2_O_2_ and other metabolites such as organic hydroperoxides and peroxynitrite are metabolised by different enzymes with peroxidase activity. Five distinct peroxidases which differ in their subcellular location and substrate specificity have been identified in trypanosomatids. Non-selenium glutathione peroxidases GPX-I (located in the cytosol and glycosome) and GPX-II (located in the endoplasmic reticulum) confer resistance against hydro- and lipid-hydroperoxides, respectively, and use glutathione and/or tryparedoxin as reducing substrates (Figure).^15^ ^,^ ^16^ Cytosolic and mitochondrial tryparedoxin peroxidase (c- or m-TXNPx) from the 2-cysteine peroxiredoxin family can detoxify peroxynitrite, H_2_O_2_, and small-chain organic hydroperoxides using tryparedoxin (Figure). Ascorbate-dependent heme-peroxidase (APX) is located in the endoplasmic reticulum and confers resistance to H_2_O_2_ challenge using ascorbate as the reducing substrate (Figure).

Other trypanothione-dependent enzymes are related to antioxidant functions, such as enzymes of the glutathione S-transferase class [which in *Leishmania* act together in elongation factor 1B (eEF1B) in the metabolism of linoleic acid hydroperoxide] and ovothiol A (a mercaptohistidine that works by eliminating H_2_O_2_ and free radicals).^14^ ^,^ ^17^ ^,^ ^18^

The molecules of the redox system are essential to protect lipids, proteins, and DNA from damage caused by oxidants. A better understanding of the molecular mediators of resistance to oxidative stress enables studying the host-parasite relationships and clarifies the mechanisms of drug resistance in the parasites.

In the next section, we describe studies of FeSODs, TXNPx, and APX, in relation to the important roles they play in defending oxidative stress and their involvement in drug resistance mechanisms in *T. cruzi* and *Leishmania* spp.

*Iron superoxide dismutase A* - FeSOD-A is an important enzyme in the antioxidant defence system that protects parasites against superoxide radicals (O_2_^−^), which are converted to oxygen (O_2_) and hydrogen peroxide (H_2_O_2_).^12^ H_2_O_2_ is metabolised by different enzymes with peroxidase activity, such as tryparedoxin peroxidase (TXN), ascorbate peroxidase (APX), peroxiredoxins (PRXs), and glutathione peroxidases (GPXs).^14^

The metalloenzyme SOD (EC 1.15.1.1) is a key component of the antioxidant defence system of many organisms and contains different metal cofactors at its active site.^19^ In trypanosomatids, SODs contain iron (Fe) in their structure. They are classified as FeSOD-A expressed in the mitochondria^20^ ^,^ ^21^ FeSOD-B1 and FeSOD-B2 are located in the glycosome,^22^ and FeSOD-C is detected in the mitochondria.^20^ Because FeSOD is absent in the human host, it can serve as a promising molecular target for drug development against trypanosomatids.

Proteomic and differential expression analyses showed that FeSOD-A is overexpressed in the *T. cruzi* population with *in vitro*-induced resistance to benznidazole.^23^ ^,^ ^24^ Molecular characterisation of the FeSOD-A gene in 25 different *T. cruzi* populations and strains showed gene amplification, increased mRNA levels, and protein expression, and FeSOD enzyme activity in a *T. cruzi* population with *in vitro*-induced resistance to benznidazole.^25^ In addition, it has been shown that parasites overexpressing FeSOD-A were more resistant to the programmed cell death stimulus resulting in cytoprotective effects.^26^

Several studies have shown the role of FeSOD-A in protecting parasites against oxidative stress. FeSOD-deficient *L. tropica* was shown to be more sensitive to oxidative stress, and FeSOD-deficient *L. donovani* has a decreased ability to infect murine macrophages.^27^ *L. amazonensis* deficient in FeSOD-A was more sensitive to oxidative stress and less effective in producing lesions in mice.^28^ In addition, mutant *L. infantum* parasites with lower levels of FeSOD-A were more susceptible to oxidative stress generated by menadione, and their ability to maintain infection in macrophages was decreased.^29^ It was demonstrated that ROS was needed for parasite infectivity, and the production of H_2_O_2_ by FeSOD-A was crucial in the process.^28^

Considering the importance of FeSOD-A for parasites and the potential use of the enzyme as a molecular target for drug development, methodologies have been used to obtain FeSOD-A knockout in *Leishmania*. Attempts to delete the FeSOD-A enzyme-coding gene using three different methodologies (conventional allelic replacement or two different CRISPR/methods) failed because *FeSOD-A* gene copies were probably retained by aneuploidy or gene amplification, suggesting that the gene plays an essential role in *L. infantum*.^29^ Similarly, *FeSOD-A* could not be deleted in *L. amazonensis*.^28^

Several studies have shown that FeSOD-A is associated with Sb^III^ activity and susceptibility to miltefosine. Tessarollo et al.^30^ reported a higher activity of FeSOD enzymes in *L. infantum* and *L. braziliensis* resistant to Sb^III^. In addition, their study observed that *L. infantum* and *L. braziliensis* became more resistant to trivalent antimony and more tolerant to oxidative stress following the overexpression of FeSOD-A.^30^ Furthermore, *L. donovani* overexpressing FeSOD-A was more resistant to miltefosine.^31^ Another study reported that a miltefosine-resistant *L. donovani* isolate overexpressed FeSOD-A and had increased enzyme activity compared with the susceptible isolate.^32^

Mutant *L. infantum* parasites with lower FeSOD-A levels were resistant to trivalent antimony and miltefosine.^29^ The transcript levels of five FeSODs (FeSOD-B1, FeSOD-B2, and three putative SODs) and six enzymes from the antioxidant defence system (ascorbate peroxidase, tryparedoxin peroxidase, peroxidoxin, non-selenium glutathione peroxidase, and NADH-dependent fumarate reductase) were evaluated to investigate whether other enzymes compensated the decrease in FeSOD-A expression. The transcript level of the enzyme ascorbate peroxidase increased in the two *FeSOD-A* ^*−/−/+*^ mutants tested.^29^ In addition, one mutant showed an increase in tryparedoxin peroxidase and SOD putative (LINF_340012900) expression, and the other had an increase in FeSOD putative SODB1, SODB2, and SOD putative (LINF_300033000) expression. The data demonstrate the deregulation of the oxidative stress defence pathways and the ability of the parasite to compensate for the lower FeSOD-A expression.

As FeSOD is not found in mammals and plays an essential role in the defence of the parasite against oxidation, it is a potential target in the development of new chemotherapeutic alternatives. Furthermore, some compounds with inhibitory effects against *T. cruzi.* Fe-SOD showed remarkable *in vitro* and *in vivo* trypanocidal activities.^33^ Benzo[*g*]phthalazine and phthalazine derivatives were more active against *T. cruzi in vitro* and *in vivo* in the acute and the chronic phase of the infection, less toxic to the host than benznidazole, and showed selective inhibitory effects on *T. cruzi* Fe-SOD enzyme activity in comparison with human CuZn-SOD.^34^ ^,^ ^35^ ^,^ ^36^ Others compounds such as polyamine macrocycles derivatives^37^ ^,^ ^38^ and tetradentate polyamines^39^ showed *in vitro* and *in vivo* activity against *T. cruzi* and selectively inhibited FeSOD of the parasite.

*Tryparedoxin peroxidase* - TXNPx belongs to the 2-cysteine peroxiredoxin family and detoxifies peroxynitrite, H_2_O_2,_ and small-chain organic hydroperoxides using tryparedoxin, a thioredoxin-related protein as an electron donor, which in turn is reduced by dihydro-trypanothione.^12^ ^,^ ^14^ TXNPx can be grouped according to their cytosolic (cTXNPx) or mitochondrial (mTXNPx) compartmentalisation. An association was found between virulence and the protein levels of both TXNPx enzyme isoforms in several *T. cruzi* strains as well as in cTXNPx-overexpressing parasites.^40^

In previous studies carried out by our group using proteomic analysis, TXNPx protein was highly expressed in the *T. cruzi* population with *in vitro*-induced resistance to BZ (17LER).^23^ We have extended the results by characterising the two TXNPx enzymes isoforms in nine other strains of *T. cruzi* that were either susceptible or naturally resistant to BZ. Our results demonstrated that cTcTXNPx and mTcTXNPx enzymes have an increased expression level in the *in vitro*-induced BZ-resistant *T. cruzi* population, contrary to what was observed in the *in vivo*-selected BZ-resistant and naturally resistant strains.^41^ Lin et al.^42^ reported a concomitant increase in the expression of cTXNP and mTXNP in arsenite-resistant *L. amazonensis.* In addition, the largest increase in cytosolic TXNPx protein levels was observed in *L. tarentolae* resistant to trivalent antimony.^43^

In *T. cruzi*, overexpression of cTcTXNPx or mTcTXNPx protected the parasite from either hydrogen peroxide or organic peroxide *t*-butyl hydroperoxide damage.^44^ ^,^ ^45^ However, parasites overexpressing either peroxidase were equally susceptible to NFX and BZ, similar to the parental control.^45^ The result may reflect an imbalance in the antioxidant defence of parasites overexpressing only one enzyme involved in the ROS detoxification pathway.

In our previous studies carried using proteomic analysis, seven protein spots corresponding to TXNPx were 2-to 5-fold more abundant in antimony-resistant *L. braziliensis* and *L. infantum* lines.^46^ Furthermore, clones from *L. braziliensis* overexpressing cTXNPx were 2-fold more resistant to Sb^III^ and more tolerant to exogenous H_2_O_2_.^47^ Previous studies have demonstrated that Sb^III^ perturbs the thiol redox potential of parasites, leading to the accumulation of ROS.^48^ ^,^ ^49^ Sb^III^ decreases the intracellular thiol buffer capacity by inducing rapid efflux of trypanothione and glutathione, and it increases the intracellular concentration of the disulphide forms of the thiols through inhibition of trypanothione reductase.^49^ The effects of Sb^III^ favour increased ROS levels. Overexpression of TXPNx confers resistance to Sb^III^ by increasing enzyme activity to reduce the ROS levels induced by exposure to Sb^III^. Data from the literature support the results, showing that overexpression of TXNPx in *L. tarentolae* causes a significant increase in resistance to Sb^III^.^43^ Wyllie et al.^50^ reported elevated levels of TXNPX in antimony-unresponsive *L. donovani* field isolates.

No difference in Sb^III^ susceptibility and a moderate resistance index to H_2_O_2_ was observed in *L. infantum* clones overexpressing cTXNPx, which could be due to different antimony-resistance mechanisms between the two *Leishmania* species analysed. Moreira et al.^51^ demonstrated that the Sb^III^-resistant *L. braziliensis* line presents an increased expression of the MRPA gene and reduced accumulation of antimony; in contrast, no difference was detected in the Sb^III^-resistant *L. infantum* line compared to their respective Sb^III^-susceptible lines.

*Ascorbate peroxidase* - APXs are class I heme-containing enzymes that catalyse H_2_O_2_-dependent oxidation of ascorbate in photosynthetic microorganisms, plants, and some trypanosomatids such as *Leishmania* spp. and *T. cruzi*; however, APX is absent in *T. brucei*.^52^ ^,^ ^53^ ^,^ ^54^ Since APX is absent in the human host and presents an important role in the antioxidant defence of the trypanosomatids, the enzyme may be considered a promising drug target for chemotherapy of the parasites.^12^ ^,^ ^53^

*T. cruzi* APX is located in the endoplasmic reticulum and forms part of the antioxidant defence system of the parasite by metabolising H_2_O_2_ to water.^53^ Furthermore, the amino acid sequence of TcAPX showed 30-35% similarity to that of plant APXs. Nogueira et al.^55^ observed that ascorbate peroxidase levels were enhanced in *T. cruzi* populations with *in vitro-*induced (17 LER) and *in vivo* selected (BZR) resistance to benznidazole. Moreover, the two BZ-resistant populations exhibited higher tolerance to exogenous H_2_O_2_ than their susceptible counterparts, and the TcAPX expression level was modulated by the stress generated by H_2_O_2_.

APX is an important factor controlling metacyclogenesis and apoptosis in *L. major*.^56^ Mukherjee et al.^57^ observed intra-chromosomal amplification of a sub-telomeric locus on chromosome 34, a region coding for APX, in antimony-resistant *L. major*. Overexpression of APX in *L. major* confers tolerance to the oxidative stress-mediated oxidation of cardiolipin, consequently protecting cells from damage.^58^ Moreira et al.^59^ demonstrated that the overexpression of APX protects *L. braziliensis* against the effects of trivalent antimony and H_2_O_2_. In addition, susceptibility tests revealed that the APX-overexpressing *L. braziliensis* lines were more resistant to isoniazid, an antibacterial agent that interacts with APX. Interestingly, this compound enhanced the antileishmanial Sb^III^ effect, indicating that the combination may be a good strategy for leishmaniasis chemotherapy. The data demonstrate that the APX enzyme is an attractive therapeutic target involved in the antimony-resistance phenotype of *L. braziliensis*, contributing to new strategies for leishmaniasis treatment.^59^

*In conclusion* - Based on our findings, *Trypanosoma cruzi* and *Leishmania* spp. are protected against oxidative stress by increasing the expression of genes that encode enzymes involved in antioxidant defence.

Our previous studies showed that *T. cruzi* population with *in vitro* induced resistance to BZ are protected against oxidative stress by a mechanism involving the overexpression of tryparedoxin peroxidase, ascorbate peroxidase, and other enzymes associated with antioxidant defence, including iron superoxide dismutase.^25^ ^,^ ^41^ ^,^ ^55^ The *T. cruzi* population with *in vivo* selected resistance to BZ presented a higher expression level of the ascorbate peroxidase protein. However, our findings revealed that the mechanisms involved in natural drug resistance in *T. cruzi* differ from those involved in induced resistance because drug resistance in *T. cruzi* is a complex process involving different parasite stages, various metabolic pathways, and the immune system of the host.

Our studies indicated that iron superoxide dismutase-A, tryparedoxin peroxidase, and ascorbate peroxidase play a significant role in antioxidant defence and in maintaining antimony resistance in *Leishmania*.^30^ ^,^ ^47^ ^,^ ^59^ Data showed that the mechanism of antimony resistance differs among *Leishmania* species. The overexpression of iron superoxide dismutase-A is involved in the Sb^III^-resistance phenotype in *L. braziliensis* and *L. infantum*;^30^ however, the overexpression of tryparedoxin peroxidase is directly associated with such phenotype in *L. braziliensis*, but not in *L. infantum*.^47^

We observed that alterations in the expression levels of enzymes important for drug resistance cause alterations in the levels of other enzymes, which can generate phenotypic compensation. In the case of a decrease in FeSOD-A in *L. infantum*, other FeSODs, and APX showed an increase in transcript levels, resulting in dysregulation of metabolic pathways related to antimony and miltefosine resistance.

The results of this study contribute to clarifying the regulation of the antioxidant defence pathway and illustrate the complexity of treating Chagas disease and leishmaniasis since the great adaptability of the parasites means that the lack of an enzyme can be overcome through changes in the expression of other enzymes in the same or similar pathways. In addition, the importance of studying the essential genes for parasites and developing new chemotherapeutic strategies using a combination of compounds that inhibit different metabolic pathways of the parasites is evident.

## References

[1] WHO (2021). Chagas disease (also known as American trypanosomiasis). https://www.who.int/news-room/fact-sheets/detail/chagas-disease-(american-trypanosomiasis).

[2] Antinori S, Galimberti L, Bianco R, Grande R, Galli M, Corbellino M (2017). Chagas disease in Europe a review for the internist in the globalized world. Eur J Intern Med.

[3] WHO (2021). Leishmaniasis. https://www.who.int/news-room/fact-sheets/detail/leishmaniasis.

[4] Burza S, Croft SL, Boelaert M (2018). Leishmaniasis. Lancet.

[5] Sales-Junior PA, Molina I, Murta SMF, Sánchez-Montalvá A, Salvador F, Corrêa-Oliveira R (2017). Experimental and clinical treatment of Chagas disease a review. Am J Trop Med Hyg.

[6] Muraca G, Berti IR, Sbaraglini ML, Fávaro WJ, Durán N, Castro GR (2020). Trypanosomatid-caused conditions state of the art of therapeutics and potential applications of lipid-based nanocarriers. Front Chem.

[7] Irigoín F, Cibils L, Comini MA, Wilkinson SR, Flohé L, Radi R (2008). Free radical biology & medicine insights into the redox biology of Trypanosoma cruzi trypanothione metabolism and oxidant detoxification. Free Radic Biol Med J.

[8] Piñeyro MD, Pizarro JC, Lema F, Pritsch O, Cayota A, Bentley GA (2005). Crystal structure of the tryparedoxin peroxidase from the human parasite Trypanosoma cruzi. J Struct Biol.

[9] Flohé L, Hecht HJ, Steinert P (1999). Glutathione and trypanothione in parasitic hydroperoxide metabolism. Free Radic Biol Med.

[10] Nogoceke E, Gommel DU, Kiess M, Kalisz HM, Flohé L (1997). A unique cascade of oxidoreductases catalyses trypanothione-mediated peroxide metabolism in Crithidia fasciculata. Biol Chem.

[11] Krauth-Siegel RL, Meiering SK, Schmidt H (2003). The parasite-specific trypanothione metabolism of Trypanosoma and Leishmania. Biol Chem.

[12] Turrens JF (2004). Oxidative stress and antioxidant defenses a target for the treatment of diseases caused by parasitic protozoa. Mol Asp Med.

[13] Krauth-siegel RL, Comini MA (2008). Biochimica et biophysica acta redox control in trypanosomatids, parasitic protozoa with trypanothione-based thiol metabolism. Biochim Biophys Acta.

[14] Castro H, Tomás AM (2008). Peroxidases of trypanosomatids. Antioxid Redox Signal.

[15] Wilkinson SR, Taylor MC, Touitha S, Mauricio IL, Meyer DJ, Kelly JM (2002). TcGPXII, a glutathione-dependent Trypanosoma cruzi peroxidase with substrate specificity restricted to fatty acid and phospholipid hydroperoxides, is localized to the endoplasmic reticulum. Biochem J.

[16] Wilkinson SR, Meyer DJ, Taylor MC, Bromley EV, Miles MA, Kelly JM (2002). The Trypanosoma cruzi enzyme TcGPXI is a glycosomal peroxidase and can be linked to trypanothione reduction by glutathione or tryparedoxin. J Biol Chem.

[17] Ariyanayagam MR, Fairlamb AH (2001). Ovothiol and trypanothione as antioxidants in trypanosomatids. Mol Biochem Parasitol.

[18] Holler TP, Hopkins PB (1990). Ovothiols as free-radical scavengers and the mechanism of ovothiol-promoted NAD(P)H-O2 oxidoreductase activity. Biochemistry.

[19] Abreu IA, Cabelli DE (2010). Superoxide dismutases-a review of the metal-associated mechanistic variations. Biochim Biophys Acta.

[20] Dufernez F, Yernaux C, Gerbod D, Noël C, Chauvenet M, Wintjens R (2006). The presence of four iron-containing superoxide dismutase isozymes in Trypanosomatidae characterization, subcellular localization, and phylogenetic origin in Trypanosoma brucei. Free Radic Biol Med.

[21] Getachew F, Gedamu L (2007). Leishmania donovani iron superoxide dismutase A is targeted to the mitochondria by its N-terminal positively charged amino acids. Mol Biochem Parasitol.

[22] Plewes KA, Barr SD, Gedamu L (2003). Iron superoxide dismutases targeted to the glycosomes of Leishmania chagasi are important for survival. Infect Immun.

[23] Andrade HM, Murta SMF, Chapeaurouge A, Perales J, Nirdé P, Romanha AJ (2008). Proteomic analysis of Trypanosoma cruzi resistance to Benznidazole. J Proteome Res.

[24] Murta SMF, Nogueira FB, Dos Santos PF, Campos FMF, Volpe C, Liarte DB (2008). Differential gene expression in Trypanosoma cruzi populations susceptible and resistant to benznidazole. Acta Trop.

[25] Nogueira FB, Krieger MA, Nirdé P, Goldenberg S, Romanha AJ, Murta SMF (2006). Increased expression of iron-containing superoxide dismutase-A (TcFeSOD-A) enzyme in Trypanosoma cruzi population with in vitro -induced resistance to benznidazole. Acta Trop.

[26] Piacenza L, Irigoín F, Alvarez MN, Peluffo G, Taylor MC, Kelly JM (2007). Mitochondrial superoxide radicals mediate programmed cell death in Trypanosoma cruzi cytoprotective action of mitochondrial iron superoxide dismutase overexpression. Biochem J.

[27] Ghosh S, Goswami S, Adhya S (2003). Role of superoxide dismutase in survival of Leishmania within the macrophage. Biochem J.

[28] Mittra B, Laranjeira-Silva MF, Miguel DC, Perrone J, Menezes B, Andrews N (2017). The iron-dependent mitochondrial superoxide dismutase SODA promotes Leishmania virulence. J Biol Chem.

[29] Santi AMM, Silva PA, Santos IFM, Murta SMF (2021). Downregulation of FeSOD-A expression in Leishmania infantum alters trivalent antimony and miltefosine susceptibility. Parasit Vectors.

[30] Tessarollo NG, Andrade JM, Moreira DS, Murta SMF (2015). Functional analysis of iron superoxide dismutase-A in wild-type and antimony-resistant Leishmania braziliensis and Leishmania infantum lines. Parasitol Int.

[31] Getachew F, Gedamu L (2012). Leishmania donovani mitochondrial iron superoxide dismutase A is released into the cytosol during miltefosine induced programmed cell death. Mol Biochem Parasitol.

[32] Veronica J, Chandrasekaran S, Dayakar A, Devender M, Prajapati VK, Sundar S (2019). Iron superoxide dismutase contributes to miltefosine resistance in Leishmania donovani. FEBS J.

[33] Beltran-Hortelano I, Perez-Silanes S, Galiano S (2017). Trypanothione reductase and superoxide dismutase as current drug targets for Trypanosoma cruzi an overview of compounds with activity against Chagas disease. Curr Med Chem.

[34] Sánchez-Moreno M, Sanz AM, Gómez-Contreras F, Navarro P, Marín C, Ramírez-Macias I (2011). In vivo trypanosomicidal activity of imidazole- or pyrazole-based benzo[g]phthalazine derivatives against acute and chronic phases of Chagas disease. J Med Chem.

[35] Sánchez-Moreno M, Gómez-Contreras F, Navarro P, Marín C, Olmo F, Yunta MJR (2012). Phthalazine derivatives containing imidazole rings behave as Fe-SOD inhibitors and show remarkable anti-T cruzi activity in immunodeficient-mouse mode of infection. J Med Chem.

[36] Olmo F, Gómez-Contreras F, Navarro P, Marín C, Yunta MJR, Cano C (2015). Synthesis and evaluation of in vitro and in vivo trypanocidal properties of a new imidazole-containing nitrophthalazine derivative. Eur J Med Chem.

[37] Sánchez-Moreno M, Marín C, Navarro P, Lamarque L, García-España E, Miranda C (2012). In vitro and in vivo trypanosomicidal activity of pyrazole-containing macrocyclic and macrobicyclic polyamines their action on acute and chronic phases of Chagas disease. J Med Chem.

[38] Olmo F, Marín C, Clares MP, Blasco S, Albelda MT, Soriano C (2013). Scorpiand-like azamacrocycles prevent the chronic establishment of Trypanosoma cruzi in a murine model. Eur J Med Chem.

[39] Olmo F, Costas M, Marín C, Rosales MJ, Martín-Escolano R, Cussó O (2017). Tetradentate polyamines as efficient metallodrugs for Chagas disease treatment in murine model. J Chemother.

[40] Piacenza L, Peluffo G, Alvarez MN, Martínez A, Radi R (2013). Trypanosoma cruzi antioxidant enzymes as virulence factors in Chagas disease. Antioxid Redox Signal.

[41] Nogueira FB, Ruiz JC, Robello C, Romanha AJ, Murta SMF (2009). Molecular characterization of cytosolic and mitochondrial tryparedoxin peroxidase in Trypanosoma cruzi populations susceptible and resistant to benznidazole. Parasitol Res.

[42] Lin Y-C, Hsu J-Y, Chiang S-C, Lee ST (2005). Distinct overexpression of cytosolic and mitochondrial tryparedoxin peroxidases results in preferential detoxification of different oxidants in arsenite-resistant Leishmania amazonensis with and without DNA amplification. Mol Biochem Parasitol.

[43] Wyllie S, Vickers TJ, Fairlamb AH (2008). Roles of trypanothione S -transferase and tryparedoxin peroxidase in resistance to antimonials. Antimicrob Agents Chemother.

[44] Finzi JK, Chiavegatto CWM, Corat KF, Lopez JA, Cabrera OG, Mielniczki-Pereira AA (2004). Trypanosoma cruzi response to the oxidative stress generated by hydrogen peroxide. Mol Biochem Parasitol.

[45] Wilkinson SR, Temperton NJ, Mondragon A, Kelly JM (2000). Distinct mitochondrial and cytosolic enzymes mediate trypanothione-dependent peroxide metabolism in Trypanosoma cruzi. J Biol Chem.

[46] Matrangolo FSV, Liarte DB, Andrade LC, de Melo MF, Andrade JM, Ferreira RF (2013). Comparative proteomic analysis of antimony-resistant and -susceptible Leishmania braziliensis and Leishmania infantum chagasi lines. Mol Biochem Parasitol.

[47] Andrade JM, Murta SMF (2014). Functional analysis of cytosolic tryparedoxin peroxidase in antimony-resistant and -susceptible Leishmania braziliensis and Leishmania infantum lines. Parasit Vectors.

[48] Mandal G, Wyllie S, Singh N, Sundar S, Fairlamb AH, Chatterjee M (2007). Increased levels of thiols protect antimony unresponsive Leishmania donovani field isolates against reactive oxygen species generated by trivalent antimony. Parasitology.

[49] Wyllie S, Cunningham ML, Fairlamb AH (2004). Dual action of antimonial drugs on thiol redox metabolism in the human pathogen Leishmania donovani. J Biol Chem.

[50] Wyllie S, Mandal G, Singh N, Sundar S, Fairlamb AH, Chatterjee M (2010). Elevated levels of tryparedoxin peroxidase in antimony unresponsive Leishmania donovani field isolates. Mol Biochem Parasitol.

[51] Moreira DS, Monte RL, Andrade JM, Santi AMM, Reis PG, Frézard F (2013). Molecular characterization of the MRPA transporter and antimony uptake in four New World Leishmania spp susceptible and resistant to antimony. Int J Parasitol Drugs Drug Resist.

[52] Raven EL (2003). Understanding functional diversity and substrate specificity in haem peroxidases what can we learn from ascorbate peroxidase?. Nat Prod Rep.

[53] Wilkinson SR, Obado SO, Mauricio IL, Kelly JM (2002). Trypanosoma cruzi expresses a plant-like ascorbate- dependent hemoperoxidase localized to the endoplasmic reticulum. PNAS.

[54] Adak S, Datta AK (2005). Leishmania major encodes an unusual peroxidase that is a close homologue of plant ascorbate peroxidase a novel role of the transmembrane domain. Biochem J.

[55] Nogueira FB, Rodrigues JFA, Correa MMS, Ruiz JC, Romanha AJ, Murta SMF (2012). The level of ascorbate peroxidase is enhanced in benznidazole-resistant populations of Trypanosoma cruzi and its expression is modulated by stress generated by hydrogen peroxide. Mem Inst Oswaldo Cruz.

[56] Pal S, Dolai S, Yadav RK, Adak S (2010). Ascorbate peroxidase from Leishmania major controls the virulence of infective stage of promastigotes by regulating oxidative stress. PLoS One.

[57] Mukherjee A, Boisvert S, Monte-Neto RL, Coelho AC, Raymond F, Mukhopadhyay R (2013). Telomeric gene deletion and intrachromosomal amplification in antimony-resistant Leishmania. Mol Microbiol.

[58] Adak S, Pal S (2013). Ascorbate peroxidase acts as a novel determiner of redox homeostasis in Leishmania. Antioxidants Redox Signal.

[59] Moreira DS, Xavier MV, Murta SMF (2018). Ascorbate peroxidase overexpression protects Leishmania braziliensis against trivalent antimony effects. Mem Inst Oswaldo Cruz.

